# Antipsychotic medications and risk of respiratory failure in the respiratory high dependency unit

**DOI:** 10.1192/bjo.2024.773

**Published:** 2024-12-04

**Authors:** Sara Winter, Tara Kirkpatrick, Karl Winckel, Faraz Honarparvar, Lewis Robinson, Timothy Tanzer, Lesley Smith, Nicola Warren, Dan Siskind, Claire Michelle Ellender

**Affiliations:** Faculty of Medicine, University of Queensland, Brisbane, Australia; Department of Psychology, West Moreton Health and Hospital Service, Queensland Health, Brisbane, Australia; Queensland Centre for Mental Health Research, Brisbane, Australia; Pharmacy Department, Princess Alexandra Hospital, Brisbane, Australia; School of Pharmacy, The University of Queensland, Brisbane, Australia; Ipswich Hospital, Ipswich, Australia; Metro South Addiction and Mental Health Service, Brisbane, Australia; Department of Respiratory & Sleep Medicine, Princess Alexandra Hospital, Brisbane, Australia

**Keywords:** Respiratory failure, antipsychotics, serious mental illness

## Abstract

**Background:**

There is a high incidence of serious mental illness (SMI) and antipsychotic use in the respiratory high dependence unit (HDU) compared with the general population. However, there is a paucity of data in the extant literature evaluating the relationships between respiratory failure and antipsychotics.

**Aims:**

To investigate the relationship between antipsychotics and respiratory failure in people admitted to a respiratory HDU, and to gain a better understanding of the potential impact of antipsychotic medications on respiratory outcomes.

**Method:**

Medical, demographic and clinical outcome data were collected for a consecutive sample of 638 individuals admitted to a respiratory HDU between the dates 1 January 2018 and 29 May 2021 at a large quaternary hospital.

**Results:**

Multivariate models controlling for confounders found that antipsychotic medications increased risk of admission for type 2 respiratory failure and chronic obstructive pulmonary disease exacerbation without hypercapnia by 3.7 and 11.45 times, respectively. For people admitted with type 2 respiratory failure, antipsychotic use increased the risk of requiring non-invasive ventilation by 4.9 times. Those prescribed an antipsychotic were more likely to be readmitted within 30 days. Over 30% of individuals were prescribed antipsychotics for an unlicensed indication.

**Conclusions:**

Poor respiratory outcomes may be a previously unknown adverse drug reaction of antipsychotics. Modifications to clinical care and clinical pathways for those with SMI prescribed antipsychotic medications, including optimising their chronic health and deprescribing where appropriate, should be prioritised.

People with schizophrenia die 16 years earlier than the general population, and at least 10% of this excess mortality gap is driven by respiratory disease.^1^ Chronic obstructive pulmonary disease (COPD) and asthma are almost twice as high in this cohort compared with the general population, and rates of pneumonia are over two and half times higher.^2^ Antipsychotics may exacerbate respiratory illness through mechanisms such as central nervous system sedation,^3^ increased rates of obstructive sleep apnoea (OSA),^4^ increased metabolic syndrome and obesity,^5^ and increased risk of aspiration due to sialorrhea.^6^ There is a paucity of data evaluating the relationship between respiratory failure and antipsychotics used for management of severe mental illness,^7^ and it is unclear whether antipsychotics may be adding risk to an already high-risk population. This is important, as respiratory failure has a high mortality rate and leads to significant morbidity in survivors.^8^ Admission to a high dependency unit (HDU) or intensive care unit (ICU) for advanced airway therapies such as non-invasive ventilation (NIV) is often required, which is costly.^9^ We aimed to investigate the relationship between antipsychotic medication use and the prevalence and severity of respiratory failure among people admitted to a respiratory HDU. The hypothesis was that people prescribed antipsychotics would be more prevalent in the HDU cohort, and that antipsychotic use would be associated with poorer outcomes including a longer HDU length of stay, longer hospitalisation and more frequent 30-day readmission rates.

## Method

The study was undertaken at the respiratory HDU at the Princess Alexandra Hospital, a large quaternary hospital for adults in Brisbane, Australia with a catchment of around 1 000 000 people. Data were extracted from digital medical records and from a prospectively collected clinical database maintained by the staff at the respiratory HDU as part of standard clinical care.

### Participants

**Fig. 1:**
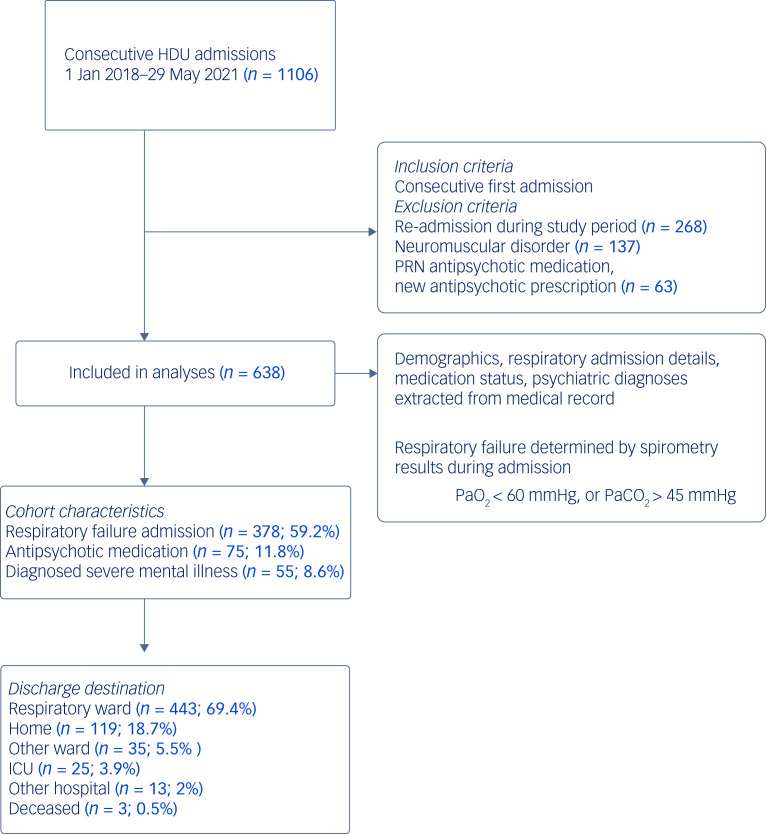
Patient flow diagram. HDU, high dependency unit; ICU, intensive care unit; PRN, psychotropic medications utilised ‘as required’; PaO_2_, partial pressure of oxygen; PaCO_2_, partial pressure of carbon dioxide.

Figure 1 outlines the patient flow of included participants in the study. Participants were consecutive admissions to the respiratory HDU between 1 January 2018 and 29 May 2021. During this timeframe there was little coronavirus (COVID-19) infection in Brisbane, Australia, so this cohort reflects pre-pandemic admission profiles. For participants with more than one HDU admission during the study period, the first admission was selected for further analysis and the other admissions excluded. Individuals admitted to the HDU with a medical history of neuromuscular disorder (specifically motor neurone disease, myotonic dystrophy, muscular dystrophy or myasthenia gravis) were excluded. Participants were categorised by indication for admission to HDU: either respiratory failure, as defined as PaO_2_ (partial pressure of oxygen) ≤ 60 mmHg, or PaCO_2_ (partial pressure of carbon dioxide) > 45 mmHg on arterial blood gas; or no respiratory failure, PaO_2_ > 60 mmHg or PaCO_2_ ≤ 45 mmHg.

Data including demographics, smoking status, respiratory, psychiatric and other medical diagnoses, respiratory admission details and psychotropic medications were extracted independently by two authors T.K. and C.M.E. Spirometry was extracted from the medical record or respiratory laboratory database. Arterial blood gas from the time of admission was recorded. Psychotropic medications utilised ‘as required’ (PRN) only were excluded because of the unknown frequency of the medication being taken, as were any new prescriptions of antipsychotic medication in the admission period. Antipsychotic medication dosage was converted to olanzapine equivalent dosage.^10^ Participants with a psychiatric diagnosis of schizophrenia or bipolar affective disorder as confirmed in the medical record were grouped as ‘serious mental illness’ (SMI) for subgroup analysis.

### Statistical analysis

Statistical analyses were conducted in SPSS version 29 for Windows. Combined means and standard deviations were calculated using standard formulations (Supplementary Data 1 available at https://doi.org/10.1192/bjo.2024.773). Group comparisons were made using *t*-tests or chi-squared tests where indicated. Multivariate relationships between antipsychotics and respiratory failure, and antipsychotics and NIV use were investigated using logistic regression analyses, and a hierarchical logistic regression model where *a priori* confounders were included. Based on clinical documentation, admission type was clustered into five groups: type 1 respiratory failure (PaO_2_ ≤ 60 mmHg with a normal or decreased PaCO_2_); type 2 respiratory failure (increase in arterial carbon dioxide PaCO_2_ > 45 mmHg with a pH < 7.35); COPD exacerbation without hypercapnia; pneumonia; or other. Multivariate relationships between antipsychotics and admission type, controlling for *a priori* confounders, were tested using multinomial logistic regression.

### Ethics

The authors assert that all procedures contributing to this work comply with the ethical standards of the relevant national and institutional committees on human experimentation and with the Helsinki Declaration of 1975, as revised in 2008. Ethics approval with a waiver of informed consent was obtained from the Metro South Health, Human Ethics Research Committee (HREC/2021/QMS/81495).

### Transparency declaration

Dr Sara Winter and project lead Dr Claire Ellender affirm that the manuscript is an honest, accurate and transparent account of the study being reported; no important aspects of the study have been omitted.

## Results

There were 638 incident admissions over the time period with 378 (59.2%) people admitted for respiratory failure and 260 (40.8%) for non-respiratory failure reasons. Of the total cohort, 54 (8.5%) participants had a history of SMI, 43 (6.7%) with schizophrenia and 11 (1.72%) with bipolar affective disorder.

### Antipsychotics prescribed

There were 75 (11.8%) participants prescribed antipsychotics at the time of admission to the HDU, of whom 50 (66.7%) had a documented SMI diagnosis, 42 (56%) with schizophrenia and 8 (10.7%) with bipolar affective disorder. Of the 75 participants prescribed an antipsychotic, 25 (33.3%) were prescribed an antipsychotic ‘off label’ with 5 (6.7%) having no documented mental health or neurocognitive diagnosis, nor acute delirium. In all, 34.7% of participants were prescribed more than one antipsychotic concurrently. The most commonly prescribed agent was olanzapine (4.4%) followed by risperidone (3.8%), quetiapine (2.5%) and aripiprazole (2.2%) (Table 1). The combined mean olanzapine equivalent dosage (OED) for the cohort prescribed antipsychotics was 11.2 mg (s.d. 7.6). Participants with SMI who were prescribed an antipsychotic (*n* = 50) had significantly higher mean total cumulative OED 18.91 mg (s.d. 10.52) than those without SMI (*n* = 25), OED 7.05 mg (s.d. 8.60), *t*(73) = −4.88, <0.001. Supplementary Table 2 displays the OED dose of participants without documented SMI.

**Table 1:** Equivalent dosages prescribed of the antipsychotic medication olanzapine

	*n* (%)^a^	Min TDD	Max TDD	Mean TDD (s.d.)	Mean OED TDD (s.d.)
First generation					
Chlorpromazine	4 (0.60)	50	400	187.5 (154.79)	6.25 (5.16)
Zuclopenthixol decanoate (intramuscular)^b^	2 (0.30)	14.29	28.57	21.43 (10.10)	15 (7.07)
Second generation					
Aripiprazole	14 (2.2)	2.5	45	22.68 (14.46)	15.12 (9.64)
Asenapine	1 (0.16)	10	10	10	10
Clozapine	7 (1.1)	225	450	278.57 (79.62)	9.29 (2.65)
Lurasidone	1 (0.16)	10	10	1.25	1.25
Olanzapine	28 (4.4)	2.5	30	8.84 (7.02)	8.84 (7.02)
Paliperidone (intramuscular)^c^	2 (0.30)	3.57	4.16	3.87 (0.29)	14.45 (1.58)
Quetiapine	16 (2.5)	50	600	284.38 (207.14)	9.47 (6.90)
Risperidone	24 (3.8)	0.25	6	4.26 (2.40)	14.20 (7.98)
Ziprasidone	1 (0.16)	80	80	80	10

Min, minimum; Max, maximum; OED, olanzapine equivalent dosage; TDD, total daily dose. All doses in are shown in mg.

a. % of whole sample (*N* = 638).

b. Two people prescribed zuclopenthixol decanoate were prescribed 200 mg and 400 mg intramuscular every 2 weeks, respectively; dose presented as daily intramuscular equivalent dose.

c. Two people prescribed long acting injectable paliperidone were prescribed 350 mg every 12 weeks and 100 mg every 4 weeks, respectively; dose presented as daily intramuscular equivalent dose.

### Respiratory failure characteristics

Demographic characteristics of the antipsychotic use cohorts are presented in Table 2. There was no significant difference in age or gender between the cohort prescribed antipsychotics compared with those not prescribed an antipsychotic. COPD (*P* < 0.001) and obesity (*P* < 0.001) were more common in the antipsychotic cohort, but there were no differences between the groups for current or past smoking status (*P* = 0.180) or OSA (*P* = 0.286).

**Table 2:** Demographic characteristics of the sample relative to antipsychotic usage

	Full sample *N* = 638	Antipsychotic *N* = 75 (11.8%)	No antipsychotic *N* = 563 (88.2%)	Test statistic	*P*-value
Gender male (%)	363 (56.9)	48 (64)	315 (56)	1.75^a^	0.186
Age, mean (s.d.)	62 (15.2) min: 18 max: 99	60.63 (16.34)	62.64 (15.03)	−1.01^b^	0.280
BMI, mean (s.d.)	31.85 (12.1) min: 12.78 max: 93.75	35.01 (11.08)	31.46 (12.18)	2.30^b^	0.020^c^
FEV1% predicted for age^d^, mean (s.d.)	57.00 (23.76) min: 11.24 max: 144.25	50.48 (25.2)	55.82 (25)	−1.21^b^	0.228
Smoking (current or past)^e^, *n* (%)	462 (72.5)	48 (67.6)	414 (75)	1.80^a^	0.180
Serious mental illness, *n* (%)	55 (8.6)	50 (66.7)	5 (0.9)	354.79^a^	**<**0.001^c^
Schizophrenia, *n* (%)	44 (6.9)	42 (56)	2 (0.4)	310.59^a^	**<**0.001^c^
Bipolar affective disorder, *n* (%)	11 (1.7)	8 (10.7)	3 (0.5)	49.14^a^	**<**0.001^c^
Medical comorbidities, *n* (%)					
COPD	276 (43.3)	48 (64)	228 (40.5)	14.89^a^	**<**0.001^c^
Obstructive sleep apnoea	132 (20.7)	12 (16)	120 (21.7)	1.14^a^	0.286
Hypertension	232 (36.4)	19 (25.3)	213 (37.8)	4.47^a^	0.035^c^
Ischaemic heart disease	97 (15.2)	12 (16)	85 (15.1)	0.04^a^	0.838
Heart failure	72 (11.3)	9 (12)	63 (11.2)	0.04^a^	0.835
Peripheral vascular disease	13 (2)	1 (1.3)	12 (2.1)	0.21^a^	0.646
Type 2 diabetes^f^	152 (23.8)	28 (37.3)	124 (22.1)	8.49^a^	0.004^c^
Admission outcomes					
Readmitted within 30 days, *n* (%)	195 (30.6)	32 (42.7)	163 (29)	5.87^a^	0.015^c^
LoS in hospital, mean (s.d.)	12.7 (24.8)	11.86 (17.65)	12.81 (25.61)	−0.31^b^	0.759
LoS in HDU, mean (s.d.)	3.29 (3.8)	2.88 (2.67)	3.34 (3.91)	−1.00^b^	0.316

min, minimum; max, maximum; BMI, body mass index; FEV1, forced expiratory volume in 1 s; COPD, chronic obstructive pulmonary disease; LoS, length of stay; HDU, high dependency unit.

a. χ^2^ test.

b. *t*-test (*P* value two-sided).

c. *P* < 0.05 significant difference between the antipsychotic and no antipsychotic groups.

d. Missing FEV1% predicted *n* = 323.

e. Missing smoking status data *n* = 15.

f. Removed gestational diabetes status *n* = 1.

A multinomial logistic regression model testing the contribution of antipsychotic use on type of admission (with *a priori* confounders of age, gender, BMI, COPD, OSA and smoking status) was significant χ^2^(28) = 190.83, *P* < 0.001, 
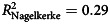
. Antipsychotic medications were a significant independent predictor of type of admission χ^2^(4) = 21.64, *P* < 0.001. This was especially so for type 2 respiratory failure and COPD exacerbation without hypercapnia, with adjusted odds ratios of 3.69 (95% CI 1.61–8.46) and 11.45 (95% CI 2.42–54.22), respectively.

For individuals prescribed an antipsychotic, there was no significant difference in total cumulative OED between those with respiratory failure (mean = 15.80, s.d. = 11.93) and without respiratory failure (mean = 12.63, s.d. = 9.49), *P* = 0.28. When total cumulative OED was classified as high (>11; *n* = 39) and low (<10; *n* = 36), there was no difference between the respiratory failure and no respiratory failure groups, χ^2^(1) = 0.54, φ = 0.08, *P* = 0.464. Because of sample size, analysis of impact of OED on admission type could not be undertaken.

People with type 2 respiratory failure and prescribed antipsychotics (*n* = 53) were more likely to require NIV during the admission (*n* = 51; 96.22%) compared with those who were not prescribed an antipsychotic (*n* = 195; 84.78%), χ^2^(1) = 4.96, φ = 0.13, *P* = 0.026 (Supplementary Data 3). For those with type 2 respiratory failure, after adjusting for age, gender, BMI, COPD, OSA and smoking status, a model testing antipsychotic medications as a predictor of NIV use during the admission was significant χ^2^(7) = 22.80, *P* < 0.002, 
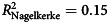
. The model correctly classified 86.6% of cases to NIV use. Antipsychotic use was a significant predictor in the model, with those prescribed an antipsychotic having over a 4.87 adjusted odds ratio of needing NIV during admission (95% CI 1.07–22.12, *P* = 0.04) (Supplementary Data 4).

### Admission outcomes

There was no significant difference in hospital length of stay, comparing those prescribed antipsychotics (mean 11.86 days) to those not prescribed an antipsychotic (12.1 days), *P* = 0.759. However, participants prescribed antipsychotics were more likely to readmit within 30 days of discharge (*P* = 0.015) compared with individuals not prescribed antipsychotic medications (Table 2).

## Discussion

In this cohort of 638 consecutive new admissions to a large, quaternary Australian hospital respiratory HDU, there was found to be a higher incidence of severe mental illness (8.5%) and antipsychotic use (11.8%), compared with the general population.^11^ The link between antipsychotic medications and respiratory illness, such as type 2 respiratory failure and COPD exacerbation, is unclear and may have multiple drivers, including a high prevalence of metabolic syndrome and obesity,^12–15^ OSA^16^ and increased incidence of smoking in those with SMI.^17–19^ We found an independent risk of antipsychotic medications when these factors were controlled for in our model. Therefore, it is possible that antipsychotics additionally exacerbate respiratory illness through other mechanisms such as central nervous system depression,^3^ potentially worsening hypoventilation and leaving those on antipsychotics more vulnerable to poor respiratory outcomes. Our results suggest that antipsychotics may increase respiratory risk in this vulnerable population.^20^ Because of limitations of the retrospective, non-randomised study design, baseline differences between the antipsychotic and no antipsychotic groups including SMI, hypertension and diabetes were not directly controlled for in the analyses, and the results should be interpreted with this in mind. Other confounding factors which result in poor respiratory outcomes were unable to be explored in this study, such as socioeconomic status, access to support and healthcare, illicit substance use, concomitant central nervous system depressants and vaccination history. People with severe mental illness experience more frequent viral infections than the general population, and vaccination rates in this population remain a significant knowledge gap.^21^ These factors may provide additional explanatory power to the relatively low *R*^2^ found in our model. Exclusion of PRN and new prescription of antipsychotic medication did not allow us to interrogate the impact of chronic versus recent/new administration on respiratory outcomes.

The OED in this cohort was lower than in previous studies^22,23^ and, contrary to these studies, OED was not associated with respiratory failure outcomes. Compared with other studies, the OED was relatively low which may have contributed to this null finding. Furthermore, the sample size for those on antipsychotics is small compared with the whole cohort, probably resulting in the wide confidence intervals in the relationship between antipsychotic use and risk of NIV use, and this may be a further contributor to the null findings regarding a dose–response relationship. The impact of OED on respiratory admission type and NIV could not be further interrogated because of sample size limitations. Moving forward, international and multicentre collaborations are required to assess large data-sets which would allow for better matching and/or controlling of potential confounds and interrogation of other clinical questions.

While the collection of large, consecutive cohort data is a strength of the study, it also poses limitations. Data were collected as part of standard care, and therefore the quality of the data is limited by clinical coding. For instance, in our data-set smoking status was not a univariate or multivariate predictor of respiratory failure. Smoking status was coded as ‘current or past’ or ‘never’ which led to a loss of nuanced relationships regarding smoking and respiratory outcomes such, as pack year, cigarettes smoked per day and years of smoking. The reasons for readmissions were not clearly articulated and therefore could not be reported here, and neither were the reasons for off-label antipsychotic use clearly articulated. The type of antipsychotic and the dosage (Supplementary Data 2) suggests that some of these individuals may have had a schizophrenia spectrum disorder diagnosis but there was a lack of clear documentation of such. Psychiatric diagnoses were reported in the clinical file based on patient self-reporting or existing annotations in clinical health and mental health files. Diagnoses of schizophrenia and bipolar disorder were not independently verified by a treating psychiatrist through clinical interview for the purposes of this study. It should also be considered that data collection (1 January 2018 to 29 May 2021) covered the period of the COVID-19 pandemic; however, because of Australian government policies and border restrictions, there were minimal or no COVID-19 related admissions in Australia until December 2021.

Despite the above limitations, our finding that 33% of participants were prescribed antipsychotic medications for off-label indications is significant. Off-label prescribing has clinical implications as well as economic implications to national prescribing budgets.^24,25^ In Australia there was a 217.7% increase in atypical antipsychotic dispensing between 2000 and 2011 led by quetiapine and risperidone, with olanzapine being the most popular prescribed atypical. In contrast, over the same period there was a 61.2% decrease in the dispensing of typical antipsychotics.^26^ Increases in atypical antipsychotic prescribing did not match the prevalence of psychotic disorders, suggesting an escalating trend for off-label use for indications other than schizophrenia or related psychoses and bipolar disorder. Antipsychotic prescribing decisions should not be taken lightly, given the known harms, including higher rates of sudden cardiac death^27^ and increased risk of death among older adults with dementia.^28^ Even at low doses (often prescribed for indications such as anxiety, agitation and insomnia)^25^ antipsychotics increase the risk for metabolic adverse effects.^29^

This study adds weight to a growing evidence base^22,23^ that poor respiratory outcomes may be an unexpected adverse drug reaction of antipsychotics. It is recommended that screening for antipsychotics be conducted as standard care in those admitted to respiratory units, or those at risk of admission. Indications for these medications should be reviewed and, if appropriate, deprescribing should be considered. Individuals with SMI should be offered vaccination against respiratory pathogens such as COVID-19, influenza and pneumococcal disease, together with smoking cessation and COPD action plans, optimisation of the prescription of inhalers and inhaler technique, nutrition support and pulmonary rehabilitation. Furthermore, those with SMI prescribed antipsychotic medications and with respiratory comorbidities would benefit from increased support from the multidisciplinary team, including nurse navigators to prevent readmissions, psychologists to work on the social and mental health determinants of health outcomes, and outreach respiratory physicians being brought into the mental health teams to manage respiratory care.

## Supporting information

Winter et al. supplementary material Winter et al. supplementary material

## Data Availability

The data that support the findings of this study are available from the corresponding author, S.W., upon reasonable request.
