# Supplementary material for: Antipsychotic medications and risk of respiratory failure in the respiratory high dependency unit
Source: BJPsych Open. 2024 Dec 4;10(6):e211. doi: 10.1192/bjo.2024.773 (PMC11698217; doi:10.1192/bjo.2024.773)
Supplement: Winter et al. supplementary material [file S2056472424007737sup001.docx]

**Supplementary materials**

*Supplementary Data S1.*

Formula for combined mean:

xc = (m.xa + n.xb)/(m = n)

xc = combined mean

xa = first group mean

xb = second group mean

m = number of items in first set

n = number of items in second set

Formula for combined standard deviation:

σ12=√(m-1)⋅σa+(n-1)⋅σb+(m⋅n)/(m+n)⋅(x2/1+x2/2-2xaxb)m+n-1

σ12 = combined standard deviation

*Table S2. Antipsychotic medications for those without SMI, with OED*

|  | **N (%) ^a^** | **Min TDD** | **Max TDD** | **Mean TDD (SD)** | **Mean OED TDD (SD)** |
| --- | --- | --- | --- | --- | --- |
| Aripiprazole | 5 (25) | 2.5 | 45 | 11.5 (16.78) | 7.67 (11.19) |
| Asenapine | 1 (4) | 10 | 10 | 10 | 10 |
| Chlorpromazine | 2 (8) | 50 | 400 | 225 (175) | 7.5 (5.83) |
| Lurasidone | 1 (4) | 10 | 10 | 10 | 1.25 |
| Olanzapine | 3 (12) | 5 | 7.5 | 5.83 (1.18) | 5.83 (1.18) |
| Quetiapine | 7 (28) | 50 | 600 | 300 (208.74) | 9.99 (6.96) |
| Risperidone | 6 (24) | 0.25 | 1 | 0.71 (0.30) | 2.36 (1.01) |
| Ziprasidone | 1  (4) | 80 | 80 | 80 | 10 |

^a^ % of those on an antipsychotic medication without a SMI diagnosis (N=25); one patient was prescribed two antipsychotics off label


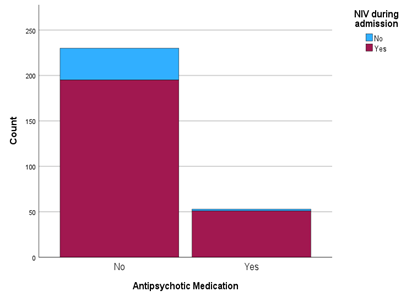


*Figure S3:  Number of patients with Type 2 Respiratory Failure requiring NIV, relative to antipsychotic medication use.*

*Table S4. Risk of NIV use for those with Type 2 Respiratory Failure on admission to HDU, adjusted for sex, age, BMI, COPD, OSA and smoking*

| **Variable** | **B (SE)** | **Wald (df)** | **p-value** | **Odds ratio** | **(95% CI)** |
| --- | --- | --- | --- | --- | --- |
| Sex (female) | 0.001 (0.38) | 0.000 (1) | 0.999 | 1.001 | (0.48-2.11) |
| Age | 0.02 (0.01) | 2.48 (1) | 0.12 | 1.02 | (0.995-1.05) |
| BMI | 0.034 (0.02) | 3.997 (1) | 0.046 * | 1.04 | (1.001-1.07) |
| COPD | 0.82 (0.397) | 4.28 (1) | 0.039 * | 2.27 | (1.04-4.96) |
| OSA | 1.18 (0.55) | 4.66 (1) | 0.03 * | 3.27 | (1.12-9.58) |
| Smoking (current or past) | -0.07 (0.46) | 0.02 (1) | 0.88 | 0.93 | (0.38-2.31) |
| Antipsychotic medication | 1.58 (0.77) | 4.21 (1) | 0.04* | 4.87 | (1.07-22.11) |

*p<.05
